# Schizophrenia and Sex Hormones: What Is the Link?

**DOI:** 10.3389/fpsyt.2020.00693

**Published:** 2020-07-15

**Authors:** Noa A. Brzezinski-Sinai, Amnon Brzezinski

**Affiliations:** ^1^ Department of Obstetrics and Gynecology, Rabin Medical Center, Beilinson Hospital, Petah Tikva, Israel; ^2^ Departments of Obstetrics & Gynecology, Hadassah-Hebrew-University Medical Center, Jerusalem, Israel

**Keywords:** schizophrenia, gonadal hormones, estrogen, progesterone, puberty, pregnancy, menstrual-cycle, menopause

## Abstract

The involvement of gonadal hormones in the pathogenesis of schizophrenia has long been suspected because the psychosis differs in women and men and the illness first makes its appearance shortly after puberty. Changes in sex hormones have been linked with increased vulnerability to mood disorders in women, while testosterone have been associated with increased sexual drive and aggressiveness in men as well as women. Some studies have found abnormal levels of estrogens and testosterone in schizophrenia patients, but the results have been inconsistent and sometimes attributed to the hyperprolactinemia effect of antipsychotics, which may interfere with sex hormones production. The purpose of this review is to present the current knowledge on the link between blood levels of sex-hormones in women during the various stages of the female reproductive life (i.e. puberty, menstrual cycle, pregnancy, contraception, and menopause) and the course of schizophrenia. We also attempt to optimize the clinical approach to women with schizophrenia at these different stages.

## Introduction

During the fertile life and reproductive aging there are significant roles for gonadal hormones in the regulation of several CNS processes, especially mood and cognitive functions. The sex hormones help to organize and activate structural connections within the brain (1). Animal studies suggest that sex steroid hormones play an essential role in myelination [e.g. (2)]. In humans, sex steroids play a developmental role in gray matter and white matter structures in the brain and may continue to exert effects in adult life and into old age (3). Cellular morphology in the brain changes throughout the life span in response to environmental stimuli, and these effects are partially mediated, levels of circulating gonadal hormones (4).

There are sex differences in the prevalence, onset, symptom profiles, and disease outcome that are evident in schizophrenia (5). There are also differences between the sexes in the association of some commonly considered risk genes for schizophrenia, such as DISC1 (6). Since sex steroids have both genomic and non-genomic actions, other factors may also be responsible for the sex differences, (e.g. the influence of sex steroids on the expression patterns of genes).

Higher brain functions, such as cognition, mood, and memory, are modulated by gonadal hormones (7). Their action is accompanied by alterations in neuron and synapse numbers, as well as in dendritic and synaptic morphology (8, 9). Although the determined sex difference in schizophrenia is relatively small, (male: female ratio 58:42 (the age of onset is earlier in males and is accompanied by more severe negative symptoms (10, 11). These observations lead many scientists to investigate a putative co-regulation between schizophrenia and gonadal steroids. As nicely described by Markham (12), the development of the central nervous system in the embryo is strongly affected by sex steroids. They modulate the interconnections between neurons. They also control the function of glial cells. Different receptor isoforms, different interactions between receptors and co-regulators, chains of events originating at the cell membrane and leading to effects in the nucleus all interact to determine selective modulations of brain cells. All these actions affect brain function which change through adolescence, pregnancy, adulthood, up to menopause and ageing (12).

In this review we summarize the current knowledge on the relationship between schizophrenia and gonadal hormones (progesterone, estrogens, and testosterone) and its clinical significance and implications. The data and recommendations are based on literature derived from searching PubMed, and PsychINFO, with appropriate search terms (schizophrenia, gonadal hormones, estrogen, progesterone, testosterone, puberty, pregnancy, menstrual-cycle, contraception menopause) for all years subsequent to the year 2000.

## Schizophrenia and Estrogen

There are well-established differences in the expression of schizophrenia in women and men (13–16) many of which have been attributed to the action of estrogen (17). Psychotic episodes are more often during periods of estrogen withdrawal, (e.g. the menstrual phase of the menstrual cycle, post-partum, following cessation of estrogen therapy, and postmenopause). Reduced relapse rates have been observed in women during pregnancy, when plasma estrogen levels are high (12, 18). Estrogens, mainly 17b-estradiol (E2), are known to exert many genomic and non-genomic effects in the CNS (19). These effects are mediated by two types of estrogen receptors, ER alpha and ER beta, and they influence neuronal development, dendritogenesis, synaptic plasticity, and neuronal excitability. The neuroprotective effects are the ones most relevant for schizophrenia. These are achieved through co-operation between membrane and genomic signals, through epigenetic mechanisms such as histone acetylation and DNA methylation, and through regulation of synaptic function, synaptic plasticity, and neurogenesis. Estrogens also promote cell survival, protecting neurons against cytotoxic insults and other forms of stress and injury (19, 20). It has been suggested that cognitive deficits in people with schizophrenia may be especially responsive to circulating estrogen levels and that cognitive performance in women with schizophrenia may be improved by estrogen (21, 22). Selective ER modulators (SERMs), such as tamoxifen, raloxifene, and bazedoxifene, bind to the ligand-binding domain of classic ERs and may act either as agonists or antagonists, depending on the tissue. In several animal models, not only estrogens but also SERMs have been shown to exert neuroprotective effects (19, 23).

### Schizophrenia and Progesterone

The role of progesterone in schizophrenia was given less consideration in the literature than estrogen. As Sun et al. (24) well described it, “existing data on progesterone in relation to schizophrenia is inconsistent, with some studies suggesting a neuroprotective role for the hormone (e.g. animal models of cognitive dysfunction and positive symptoms), while other studies posit a disruptive impact of the hormone (e.g. negative correlations with symptom modulation in patients). Based on the clinical studies available there appears to be a link between lower symptom scores and the mid-luteal phase of the menstrual cycle, which is associated with high progesterone/high estradiol levels” (24). There are some animal studies which support the inhibiting effect of progesterone on hyperactive behavior (25).

Recently, it was reported that baseline levels of progesterone were significantly higher in first-episode antipsychotic-naïve patients with schizophrenia than in normal controls. It was speculated that lower levels of progesterone at baseline may predict better therapeutic outcome of antipsychotic treatment (26).

### Schizophrenia and Testosterone

The earlier age of onset and greater incidence of schizophrenia among males might be partially explained (apart from the “estrogen hypothesis”) by testosterone exposure (12). However, Most of the information about testosterone and schizophrenia is in reference to males. Elevated levels of testosterone have been associated with increased psychiatric symptoms (27). Very few studies examined testosterone serum levels in men with schizophrenia. Some of them reported either lower testosterone levels compared to healthy controls (28) or no difference between these groups (29).

Hyperprolactinemia often follows long-term antipsychotic drug use, especially the typical antipsychotics (e.g. Risperidone), so the studies of gonadal hormone levels might be affected by the abnormal prolactin levels (30). It should be pointed out that hyperprolactinemia is less common with the newer antipsychotics (e.g., clozapine and aripiprazole), and thus does not occur so often anymore.

While one study has reported normal testosterone levels for unmedicated, first episode patients (31), others (32) reported that gonadal hormone levels among men acutely admitted (for symptom exacerbation or at first episode) were significantly lower than controls (both testosterone and estrogen). It has also been reported that circulating testosterone levels are negatively correlated with negative symptoms (33).

Regarding women, it was recently reported (34) that there were statistically significantly higher levels of serum DHEA-S in schizophrenic women than in normal controls. No statistically significant difference was determined between the groups regarding serum testosterone and cortisol levels. It was suggested that DHEA-S (and not testosterone) might be a potential biologic marker for schizophrenia in women. However, further research with greater patient numbers is required to verify this theory.

## Clinical Implications

Our cumulative knowledge about the effects of gonadal hormones on brain functions has significant clinical implications in every stage of the schizophrenic woman’s reproductive life-cycle. The following is an attempt to optimize the approach to these women at each of these stages (i.e., puberty, menstrual cycle, pregnancy, contraception, and menopause).

### Puberty

In women, but not man, there is significant inverse relation between puberty and age at onset of schizophrenia. This difference led to the theory that female hormones act on the developing brain to protect its function and delay the expression of psychosis (35). Nevertheless, it should be remembered that the start of puberty is associated with more than hormonal changes. It is the beginning of increasingly divergent psychosocial pathways that differentiate women and men at this important time in their lives. Therefore, special attention should be made by the family physicians as well as family members and teachers, to possible early signs of schizophrenic behavior. It should be taken into account that stress in adolescent girls has been implicated in increased prevalence of depression and anxiety disorders. Integrative clinical approach is suggested while examining pubertal psychiatric complaints and genetical and psychosocial aspects should be taken into consideration (36).

### The Menstrual Cycle

The severity of psychotic symptoms in pre-menopausal women with schizophrenia increases in phases of the cycle with low estrogen (37–41). Also, a negative correlation has been reported between estrogen levels and the required dose of antipsychotics in menstruating women (42). Levels of estrogen are typically peaking around the time of ovulation (midcycle) and declining before the start of menses. The estrogen protection hypothesis predicts that psychotic disorders worsen at times in the cycle when estrogen is low, around menstruation (43) and several publications support this assumption. A recent meta-analysis of studies with women with psychiatric diagnoses demonstrated worse mental health outcomes around the time of menstruation (44).

Psychiatric admission rates are higher than expected during the perimenstrual phase. This is in agreement with the observation that a worsening of psychotic symptoms occurs during this phase (45). Most of the studies about the menstrual cycle and psychotic symptoms lack measurements of hormonal fluctuations throughout the menstrual cycle. Therefore, further research with more precise measurements of the menstrual cycle and symptomatology is required.

### Pregnancy

Typically the age of onset for schizophrenia in women is during the childbearing years so many of these women become pregnant. Pregnancy reportedly appears to worsen mental symptoms in women with schizophrenia. Psychotic denial of pregnancy is a symptom that poses especially high risks for poor outcomes if not addressed. Up to now, little research has been done into interventions for psychotic disorders in pregnancy and in particular, few studies have been done into use of antipsychotic medication (46, 47). Research on the safety of medication during pregnancy and breastfeeding is limited. Nevertheless, it is still necessary to make treatment recommendations based on the accumulated current information. It is generally accepted that there is a greater risk for the mother and the fetus in not treating schizophrenia during pregnancy and postpartum than in providing antipsychotic treatment (48).

The following are the main recommendations to care providers suggested in the literature:

“Take a sexual history and initiate discussion about intimate relationships and contraception with all women diagnosed with schizophrenia. During pregnancy, adjust antipsychotic dose to clinical status, link the patient with prenatal care services, and help her prepare for childbirth. There are pros and cons to breastfeeding while on medication, and these need thorough discussion. During the postpartum period, mental health home visits should be provided. Parenting support is critical” (46, 47).

“Psychoeducation can apparently reduce pregnancy complications for women with schizophrenia. Short-term, focused psychotherapy can be helpful for some pregnant women with schizophrenia. Some modifications need to be made in the inpatient treatment of pregnant patients with schizophrenia. In the postpartum period, women can be especially susceptible for acute exacerbation of their schizophrenia” (49).

### Contraception

It has been reported that mental illness is a risk factor for inconsistent contraceptive use (50). The prevalence of sexually transmitted infections is high in this population. The overall rate of pregnancy in women with schizophrenia of child-bearing age is lower than in the general population, but the percentage of unwanted pregnancies is higher than that in the general population. Contraceptive counseling to women and their partners should be part of the care for women with schizophrenia. Women with schizophrenia, who smoke, are overweight, have diabetes, migraine, cardiovascular disease or thrombophilia, and should be offered non-hormonal contraception. Women with more than one sexual partner should be advised on barrier methods in addition to any other contraceptive measures they are using. Long-acting contraceptives, such as intrauterine devices and progesterone-depot injections, are reasonable options for schizophrenic women. Women who completed their family planning might be offered tubal ligation (or more recently salpingectomy) [for further information and discussion see (51)].

### Menopause

Women with schizophrenia may have the same climacteric complaints as healthy women (e.g. vasomotor, physical, cognitive, sexual, and psychosocial symptoms) (52, 53). These symptoms are sometimes aggravated by factors associated with schizophrenia such as lack of occupation, poverty, substance abuse, loneliness, and side-effects of antipsychotic medications. Moreover, the psychotic symptoms of schizophrenia, such as hallucinations and delusions, worsen as women approach the menopause, while in men, at the same age, these symptoms generally improve (52, 53).

It has been reported that many schizophrenia women (and their relatives) perceive menopause as being associated with increased psychiatric symptoms (52, 54) and a decreased quality of life (52, 55). The main symptoms that worsen in these women are depression, anxiety, fatigue, and poor memory (53).

Apart from vasomotor symptoms many women with schizophrenia suffer also from associated symptoms, such as insomnia, irritability, and subsequently reduced quality of life. Estrogen therapy with or without a progestogen is the proven most effective treatment (56–58). Treatment of moderate to severe vasomotor symptoms remains the primary indication for HT (hormonal treatment). Almost all systemic HT products have government approval for this indication. Selective estrogen receptor modulators (SERM’s) such as tamoxifen, raloxifene, or bazedoxifene, on the other hand can make flushing worse. As with women in the general population, HT should be offered early after the start of menopause, and potential hazards, such as breast and cardiovascular changes, need to be monitored.

Women with schizophrenia at menopause may require increased antipsychotic doses. The need for higher doses may continue and become more marked the longer the period since menopause (59, 60). Drugs that raise prolactin levels are best avoided.

## Conclusions

The data presented above indicate that the gonadal hormones are involved in the pathogenesis of schizophrenia and affect its course. A clear link between sex hormones and schizophrenia is based on numerous studies and clinical observations of later onset of schizophrenia associated with early puberty in girls, lower relapse of psychiatric symptoms during pregnancy, high relapse postpartum, fluctuation of the symptoms across the menstrual cycle, and exacerbation of psychotic symptoms in women with schizophrenia during the menopausal transition. However, the exact mechanism by which sex hormones affect the appearance, course and outcome of schizophrenia is still not entirely understood.

A medical and social approach to women with schizophrenia should be based on our knowledge about the inter relationship between these women and their sex hormones in every stage of their reproductive life.

## Author Contributions

The authors contributed equally to the literature search and the writing of the manuscript.

## Conflict of Interest

The authors declare that the research was conducted in the absence of any commercial or financial relationships that could be construed as a potential conflict of interest.
